# Supplementary material for: Effectiveness of brief mindfulness intervention for college students’ problematic smartphone use: The mediating role of self-control
Source: PLoS One. 2022 Dec 22;17(12):e0279621. doi: 10.1371/journal.pone.0279621 (PMC9778502; doi:10.1371/journal.pone.0279621)
Supplement: Attachment — Submitted filename: Response to Reviewers.docx [file pone.0279621.s002.docx]

**List of Responses**

Dear Editors and Reviewers:

Thank you for your letter and for the reviewers’ comments concerning our manuscript entitled “Effectiveness of Brief Mindfulness Intervention for College Students’ Smartphone Addiction: The mediating Role of Self-control”. Those comments are all valuable and very helpful for revising and improving our paper, as well as the important guiding significance to our researches. We have studied comments carefully and have made correction which we hope meet with approval. Revised portion are marked in red in the paper. The main corrections in the paper and the responds to the reviewer’s comments are as flowing:

Responds to the reviewer’s comments:

In this study, correlation analyses, t-tests and ANOVAs were conducted using the subscores as well as the total scores of MPATS and SCS, but the authors made no reference to the results of those subscores in Results and Discussion sections. Moreover, the authors did not use any of those subscores in a mediation analysis. Therefore, I recommend the authors to use only the total scores of MPATS and SCS as the problematic smartphone use score and the self-control ability score, respectively. In that case, the removal of the subscore results of MPATS and SCS from Table 1-3 is also needed. Besides, it would be better to list not only pre-intervention variables but also post-intervention variables and demographic variables (i.e., gender and age) in Table 1.

Response: It is really true as Reviewer said that we made no results of subscores in Results and Discussion sections because they were not the focus of our research. Thus, as you suggested, we have removed all subscore results in our paper.

Moreover, demographic variables were compared in table 1. Thus, as you suggested, we have listed pre-intervention variables, post-intervention variables and demographic variables in Table 2.

**Reviewer 1:**

1. The abstract should indicate whether the recruitment is random.

Response: It is really true as Reviewer suggested that we should indicate whether the recruitment is random, and now we have made correction in abstract according to your comments.
2. Smartphone addiction is a worldwide problem, and it is better to mention the global trend of it in the introduction section.
Response: Thank you for your comment. Now we have mentioned the global trend of smartphone addiction in the introduction section.
3. "Psychotherapy is defined as the treatment of mental health symptoms or disorders or problems of living, and/or facilitation of personal growth by psychological means", the source should be provided here.
Response: We are very sorry for our negligence, and as you suggested, the source was provided here.
4. "Although previous studies have preliminarily confirmed the effect of a mindfulness intervention on smartphone addiction, its mechanism has not been fully revealed", more recent research is needed here to further clarify your conclusions. What are the existing mechanisms and what needs to be supplemented?
Response: We are very sorry for our negligence, and we have re-written this sentence. “Most existing studies focused on the mediating effect of trait mindfulness, rather than the mediating mechanism of mindfulness intervention. Therefore, it is necessary to further supplement the mediating mechanism of mindfulness training on mobile phone addiction”.

5. What are the research gaps and significance in your study? It should be stated separately and clearly.
Response: It is really true as Reviewer suggested that the research gaps and significance should be stated separately and clearly. Thus, now we have made a supplement of these sections and re-written our hypothesis in the end of the Introduction.

6. Please use more accurate intervention terms, such as "at baseline" instead of "before the experiment proper".
Response: We are very sorry for our incorrect writing, now we have replaced “before the experiment proper” with “at baseline”, and examined other mistakes.
7. Why is there no separate table reporting demographic information in the results section? You also need to clarify whether the demographic information has potential risk of bias.
Response: We are very sorry for our negligence, and the separate table reporting demographic information was showed at results section now. The demographic variables were also compared in table 1.
8. You'd better check whether the statements in the background and discussion sections have sources. And please update your citations, citing the latest research results as much as possible.

Response: Thank you for your comment. Now, all sources were provided in the background and discussion sections. We also updated our citations, and citing the latest studies as much as possible.

**Reviewer 2:**

Abstract:
This sentence states, "We examined the effects of a brief mindfulness intervention on smartphone addiction and investigated if this effect is moderated by self-control." However the authors undertook a mediation analysis. Please clarify.

Response: We are very sorry for our incorrect writing, now we have replaced “moderated” with “mediated” in abstract section.

Introduction:
I would argue the rationale for hypothesis 1 and Hypothesis 2 are lacking. The authors suggest and credit prior research suggesting mindfulness intervention can alleviate smartphone addiction. Then, what does Hypothesis 1 add to the literature, beyond a replication in a new sample? Similarly, the authors state mindfulness interventions can improve self-control as part of their literature review, but their hypothesis suggests mindfulness training will improve self-control in the sample. Wouldn't we assume this already, based on what is stated we know? A clearer statement of what this study would add to the literature would help prove its worth.
Response: It is really true as Reviewer suggested that a clearer statement of what this study would add to the literature is needed. The majority of previous studies had focused on the effects of long-term mindfulness training on self-control and smartphone addiction, while a few intervention studies had explored the relationship between them for college students; thus, the adoption of brief mindfulness training in this study is purposeful and fills a missing research gap. Moreover, previous studies have examined the changes in smartphone addiction and self-control before and after mindfulness intervention. However, the mediating mechanism by which mindfulness training improves smartphone addiction has rarely been explored. Thus, now we have made a supplement of these sections and re-written our hypothesis in the end of the Introduction.

Methods:
It appears Chronbach's alpha for the MPATS was .65 for the pre-intervention intervention assessment, representing poor internal consistency. Why do the authors think this is so?
Response: Thank you for your comment. In our assessments, .65 was not the Chronbach's alpha for the MPATS, while it was the alpha score for a subscale of MPATS. As the Reviewer suggested, we have removed all subscore results in our paper. And the alpha for the MPATS was .91, which means that there was acceptable internal consistency reliability for MPATS.

Please state Chronbach's alpha for the SCS.
Response: We are very sorry for our negligence, and the Chronbach's alpha for the SCS has been supplemented now.

Is a sample size of 48 enough to detect a mediated effect? Even for a reported large effect size for mindfulness, this appears a low size to detect a complicated effect such as mediation. Was the power analysis undertaken with the mediation analysis in mind, specifically? And does g*power handle mediation? Clarification that the power is appropriate for mediation is warranted, as there is possibility of Type 1 error. The authors may find the following references useful:
Schoemann, A. M., Boulton, A. J., & Short, S. D. (2017). Determining power and sample size for simple and complex mediation models. Social Psychological and Personality Science, 8(4), 379-386.
Fritz, M. S., & MacKinnon, D. P. (2007). Required sample size to detect the mediated effect. Psychological science, 18(3), 233-239.
Response: Thank you for your comment. As you said, G*power can’t handle mediation sample size. While in our study, we used G*power to only determine the sample size of brief mindfulness training on smartphone addiction.

Moreover, in the previous questionnaire survey research, the number of subjects in the mediation effect test needs to reach a large sample size. However, in experimental research, it is impossible to complete the intervention of a large sample size. Therefore, according to previous studies (Josefsson, T. et al. (2019). Effects of Mindfulness-Acceptance-Commitment (MAC) on Sport-Specific Dispositional Mindfulness, Emotion Regulation, and Self-Rated Athletic Performance in a Multiple-Sport Population: an RCT Study. *Mindfulness*), we recruited 48 subjects in our research. The PROCESS was used to test the mediating effect of self-control between mindfulness intervention and smartphone addiction, The number of Bootstrap samples was 5000.

Discussion:
5th paragraph of the discussion mentions self-control in terms of inhibiting 'animal impulses' and 'deviant behavior,' which is out of scope of the current topic. Smartphone use is quite normalized, with many people self-reporting 'addiction' to their devices, suggesting this behavior is likely not entirely abnormal or 'deviant.' The authors would improve their work by fitting in their findings within more recent literature of addictive behaviors, such as below:
Brand, M., Wegmann, E., Stark, R., Müller, A., Wölfling, K., Robbins, T. W., & Potenza, M. N. (2019). The Interaction of Person-Affect-Cognition-Execution (I-PACE) model for addictive behaviors: Update, generalization to addictive behaviors beyond internet-use disorders, and specification of the process character of addictive behaviors. Neuroscience & Biobehavioral Reviews, 104, 1-10.
Response: We are very sorry for our incorrect writing, and thank you for your comment. We have re-written this part, which aims to explain more clearly about the mediating role of self-control.

Minor:
Some of the grammar and syntax throughout the paper, especially in the introduction section, could be improved. Some sentences read awkward to the reader.

Response: Thank you for your comment. We have examined the grammar and syntax throughout the paper, and re-written the most parts of introduction section in our paper.

The phrases of "smartphone addiction" or referencing smartphones as an addictive disorder could be rephrased to something like "problematic smartphone use,' given the debate in the field whether this behavior can be classified as a true 'addiction.'

Response: Thank you for your comment. As you suggested, it is debatable whether this behavior can be classified as a true 'addiction'. And the tool we use can only measure the tendency of smartphone addiction. Therefore, we have replaced “smartphone addiction” with “problematic smartphone use”.

Special thanks to you for all your good comments!

We tried our best to improve the manuscript and made some changes in the manuscript. These changes will not influence the content and framework of the paper. And here we did not list the changes but marked in red in revised paper.

We appreciate for Editors/Reviewers’ warm work earnestly, and hope that the correction will meet with approval.

Once again, thank you very much for your comments and suggestions.
