# Supplementary material for: Effectiveness of brief mindfulness intervention for college students’ problematic smartphone use: The mediating role of self-control
Source: PLoS One. 2022 Dec 22;17(12):e0279621. doi: 10.1371/journal.pone.0279621 (PMC9778502; doi:10.1371/journal.pone.0279621)
Supplement: Attachment — Submitted filename: Response to Reviewers.docx [file pone.0279621.s003.docx]

**List of Responses**

Dear Editors and Reviewers:

Thank you for your letter and for the reviewers’ comments concerning our manuscript. Those comments are all valuable and very helpful for revising and improving our paper, as well as the important guiding significance to our researches. We have studied comments carefully and have made correction which we hope meet with approval. Revised portion are marked in red in the paper. The main corrections in the paper and the responds to the reviewer’s comments are as flowing:

Responds to the reviewer’s comments:

The authors should italicize statistical symbols and add the value of degrees of freedom. In the results of independent-sample t-tests, the p-value of FMI seems wrong. Table 3 does not include "group," one of the independent variables. The authors should insert a space between words or after commas in many sentences, such as L28, L180, L197, L311, L315, L317, and L318. It would be better to change "fourteen items" to "14 items" in L142 and "applied by" to "applied to" in L110.

Response: Thank you for your comments. Firstly, we are very sorry for our incorrect writing, and we have italicized statistical symbols, added the value of degrees of freedom, and rewritten the p-value of FMI. Secondly, we have inserted a space between words or after commas in above sentences, and we have also changed "fourteen items" to "14 items" and "applied by" to "applied to". Finally, in table 3, the result of “group” was same as “direct effect”, thus it did not include "group" to avoid duplication. In addition, according to the suggestion of Reviewer 3, we presented the ANOVA results in table 3.

**Reviewer 2:**

I would recommend stating the mediation effect as more of a preliminary finding in need of further replication using a larger sample. When the sample size is this small, applying mediation is more of an 'exploratory' route.

Response: It is really true as Reviewer suggested that our sample size was relatively small. Thus, in future research, the mediation effect should be further replicated using a larger sample size. Now we have rewritten the limitation part in the end of the Discussion.

I would also recommend stating what the participants actually did during the mindfulness exercise? Activities, meditation, etc. are mentioned but not described.

Response: Thank you for your comments. As reviewer recommend, we have stated what the participants actually did during the mindfulness exercise in “2.3 Procedure” part.

**Reviewer 3:**

for the ANOVA result, you shall present it with table.

Response: Thank you for your comments. As reviewer recommend, we have presented the ANOVA results in table 3.

it is not a 2x2 factorial design. I guess it is a quasi-experimental only. given the above study design, you should use general linear model instead of 2-way repeated measure ANOVA for data analysis. You better consult a statistician for the correct test used.

Response: Thank you for your comment. In our study, the group (mindfulness group vs. control group) was a between-subject factor, and the time (pre-intervention vs. post-intervention) was a within-subject factor, thus it could been seen as a 2×2 mixed factorial design. And 2-way repeated measure ANOVA should be used for data analysis, and previous studies provided evidence for this (Zhang, C.-Q., Si, G., Duan, Y., Lyu, Y., Keatley, D. A., & Chan, D. K. C. (2016). The effects of mindfulness training on beginners’ skill acquisition in dart throwing: A randomized controlled trial. Psychology of Sport and Exercise, 22, 279–285.).

We tried our best to improve the manuscript and made some changes in the manuscript. These changes will not influence the content and framework of the paper. And here we did not list the changes but marked in red in revised paper.

We appreciate for Editors/Reviewers’ warm work earnestly, and hope that the correction will meet with approval.

Once again, thank you very much for your comments and suggestions.
